# Pediatric rhabdomyosarcoma incidence and survival in the United States: An assessment of 5656 cases, 2001–2017

**DOI:** 10.1002/cam4.5211

**Published:** 2022-09-07

**Authors:** Matthew T. McEvoy, David A. Siegel, Shifan Dai, Mehmet Fatih Okcu, Mark Zobeck, Rajkumar Venkatramani, Philip J. Lupo

**Affiliations:** ^1^ Texas Children's Cancer Center, Baylor College of Medicine Houston Texas USA; ^2^ Division of Cancer Prevention and Control Centers for Disease Control and Prevention Atlanta Georgia USA; ^3^ CyberData Technologies, Inc. Herndon Virginia USA

**Keywords:** rhabdomyosarcoma, epidemiology, pediatric cancer, incidence, survival, soft tissue sarcoma

## Abstract

**Background:**

While rhabdomyosarcoma (RMS) is the most common soft tissue sarcoma in children and adolescents, past epidemiology studies of this malignancy used data that covered <30% of the US population. Therefore, we evaluated RMS incidence using data from U.S. Cancer Statistics (USCS) and survival trends using the National Program of Cancer Registries (NPCR), which covers 100% and 94% of the U.S. population, respectively.

**Methods:**

Incidence and survival were assessed for pediatric patients diagnosed with RMS during 2003–2017 and 2001–2016, respectively. Both demographic and clinical variables were evaluated. Age‐adjusted incidence rates, average annual percent change (AAPC), and 5‐year relative survival (RS) were calculated, all with corresponding 95% confidence intervals (CIs). Cox regression models were used to evaluate the impact of demographic and clinical variables on survival.

**Results:**

We identified 5656 primary RMS cases in USCS during 2003–2017. The age‐adjusted incidence rate was 4.58 per 1 million (95% CI: 4.46–4.70) with an AAPC of 0.3% (95% CI: −0.7 to 1.2%). In NPCR, 5‐year RS for all cases was 68.0% (95% CI: 66.6–69.3%). In multivariable analyses, non‐Hispanic (NH) Black cases had worse survival compared with NH White cases (hazard ratio [HR] = 1.16, 95% CI: 1.01–1.33).

**Conclusion:**

The incidence and survival rates were stable in the largest and most comprehensive population‐based analysis for pediatric RMS cases in the U.S. Additionally, we observed a survival disparity among NH Black cases. Findings from this study could inform interventions to address disparities, risk stratification strategies, and clinical trial design.

## Graphical abstract

In a large population‐based analysis of pediatric rhabdomyosarcoma cases in the U.S. from 2001‐2017, the incidence and survival rates were stable compared to previous studies. Additionally, a novel survival disparity among Black cases was observed.

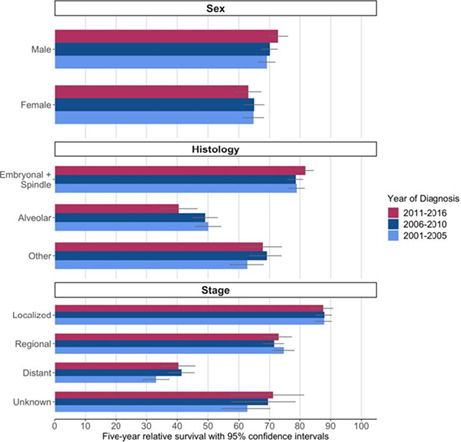

## 1 INTRODUCTION

Rhabdomyosarcoma (RMS) is a malignant tumor that originates from developing skeletal muscle cells. The most common soft tissue sarcoma in children and adolescents, RMS represents about 3–4% of all pediatric cancers, which corresponds to about 350 new cases diagnosed annually in the United States.^1^ Both incidence and survival for RMS have been characterized by several different demographic and clinical factors. Historically, the two major histologic subtypes are embryonal (ERMS) and alveolar (ARMS), which make up about 70% and 20% of RMS cases, respectively.^2^ Recently, due to advances in cytogenetic testing, the RMS subtype is now primarily classified according to the presence or the absence of a fusion involving the *FOXO1* gene.^3^ These pathological characteristics and other clinical factors—including patient age; tumor histology, size, and primary anatomic site; and the presence of metastases—have been confirmed as valid prognostic factors through both epidemiological studies and clinical trials.^4^, ^5^, ^6^, ^7^ As such, they have been incorporated into staging protocols and risk‐adapted therapy strategies in successive clinical trials from national cooperative groups.^1^, ^8^, ^9^ Sociodemographic variables such as sex, race/ethnicity, and economic status have also been explored in several studies, with some emerging patterns but overall varying results.^10^, ^11^, ^12^, ^13^, ^14^ Unfortunately, despite initial improvements in outcomes during the 1970s and 1980s, overall survival rates in the U.S. have subsequently plateaued and recent clinical trial interventions have failed to demonstrate success for patient populations across the spectrum of low‐, intermediate‐, and high‐risk disease.^3^, ^15^, ^16^, ^17^, ^18^, ^19^

While there have been notable registry‐based studies of pediatric RMS, including those using data from the Surveillance, Epidemiology, and End Results (SEER) Program covering <30% of the U.S. population^5^, ^16^, ^20^, ^21^, ^22^, ^23^ as well as international registries,^24^, ^25^ there are still considerable gaps in our understanding of the overall epidemiology of this clinically important malignancy. Specifically, more comprehensive and contemporary data are needed to characterize (1) incidence patterns of RMS to inform new studies evaluating the etiologies of these tumors and (2) survival trends of RMS to inform new risk stratification strategies and novel therapies. Therefore, the purpose of this study is to provide an updated and more comprehensive investigation of incidence and survival for pediatric RMS using population‐based databases from U.S. Cancer Statistics (USCS) and the National Program of Cancer Registries (NPCR), which includes data from 100% and 94% of the US population, respectively.

## 2 METHODS

### 2.1 Data source and study population

The USCS database was used to assess incidence between 2003 and 2017, whereas the NPCR survival database was used to evaluate survival for patients diagnosed between 2001 and 2016.^26^ The USCS database includes all 50 states and the District of Columbia (DC), encompassing 100% of the US population. The NPCR survival database includes all states and DC except for Connecticut, Hawaii, Indiana, Iowa, Kansas, and New Mexico, covering 94% of the US population. The NPCR conducts active case follow‐up or linkage with the CDC's National Death Index.

The International Classification of Childhood Cancer, third edition (ICCC‐3) was used to define RMS cases for all individuals diagnosed at <20 years of age.^27^ Within ICCC category IXa (RMS), we identified relevant International Classification of Disease for Oncology, third edition (ICD‐O‐3) morphology codes and included the following RMS subtypes in our analysis: 8900/3 (rhabdomyosarcoma NOS), 8902/3 (mixed‐type), 8910/3 (embryonal), 8912/3 (spindle cell), 8920/3 (alveolar), and 8991/3 (embryonal sarcoma).^28^ Due to low numbers of cases (*n* < 15) and inconsistent inclusion in prior pediatric epidemiology studies, subtypes 8901/3 (pleomorphic RMS, adult type) and 8921/3 (RMS with ganglionic differentiation) were excluded. Only first‐primary, malignant tumor cases with microscopic diagnosis confirmation were included. Cases determined by autopsy or death certificate only were excluded.

### 2.2 Variables

Incidence and survival rates were reported overall and according to clinical and sociodemographic variables. Tumor histologies included embryonal, spindle cell, and alveolar; additionally, we created an “other” category to represent morphology codes 8900/3, 8902/3, and 8991/3, both to increase the sample size for ease of comparison, and because these morphologies are largely considered outdated.^29^ Anatomic site of the primary tumor was classified into eight categories according to the definitions in the open Children‘s Oncology Group (COG) clinical trial (refer to Appendices III, V, and VI).^30^ Sites considered clinically favorable include orbit, head/neck (excluding the para‐meningeal region), biliary tract/liver, and genitourinary system (excluding bladder/prostate). Unfavorable sites include the para‐meningeal region, bladder/prostate, extremity, and trunk/other (including retroperitoneum and unknown). To characterize the extent of the disease, we utilized the merged summary stage.^31^ Finally, a chronological variable that divided the survival data into 5‐ or 6‐year time frames was generated to examine any potential changes in outcomes over time.

Patients were further stratified by sociodemographic characteristics that included sex, race/ethnicity, age at diagnosis, and socioeconomic status (SES) as well as the geographic census region and population density of primary residence by county. Age was categorized into known prognostic delineations for pediatric RMS based on previous reports.^4^ Race/ethnicity was grouped into non‐Hispanic (NH) White, NH Black, NH Asian‐Pacific Islander (API), NH American Indian/Alaska Native (AI/AN), NH All Other Races Combined, and Hispanic. For survival analysis, the NH All Other Races Combined group was not available and 63 NH Unknown cases were excluded. SES was grouped into five categories as defined by the Appalachian Regional Commission index‐based county economic classification system and was regrouped into three categories (top 25%, 25–75%, and bottom 25%) as has been described previously.^32^

### 2.3 Statistical analysis

Incidence rates (IRs) for each variable were expressed per 1 million persons and age‐adjusted to the 2000 U.S. standard population. Incidence rate ratios (IRRs) were reported for each variable, generally with the largest and/or the most clinically favorable group as the referent. Temporal trends in incidence were described using average annual percent change (AAPC) calculated by joinpoint regression.^33^ A maximum of two joinpoints were used to determine a change in direction of the trend.

In the survival analyses, outcomes were measured using 5‐year relative survival (RS), which aims to represent cancer survival in the absence of other causes of death, or cancer‐specific survival. RS is defined as the ratio of the observed survival of patients with cancer to the expected survival of a matched cohort of cancer‐free individuals. RS is calculated using expected life tables that are stratified by age, sex, race/ethnicity, SES, geographic location, and calendar year of diagnosis; the methods of estimation have been described elsewhere.^34^ All‐cause survival curves, overall and by demographic and clinical variables, were generated using the Kaplan–Meier method. Statistical testing for survival curves was performed using the log‐rank test. Univariate and multivariable Cox regression models were conducted to examine the effects of demographic and clinical variables on 5‐year all‐cause survival. Hazard ratios (HR) were generated for each variable, with a higher HR between compared groups indicating a higher risk of death. Variables were included in the multivariable model if their univariate p value was <0.20. For survival curves and regression analysis, NH API and NH AI/AN groups were combined into an “NH Other” grouping.

The SEER*Stat 8.3.8 software program was used to perform all analyses related to incidence and relative survival.^35^ All‐cause survival curves and related Cox regression models were generated using SAS Version 9.4.^36^ All tests were two‐sided and a *p* value of 0.05 was considered statistically significant. The data that support the findings of this study are available upon request by contacting uscsdata@cdc.gov.^26^ The data are not publicly available due to privacy and legal restrictions.

## 3 RESULTS

### 3.1 Incidence

In the USCS database, for the period 2003–2017, 5656 cases of RMS were diagnosed in children and adolescents <20 years of age (Table 1). The overall incidence for this time period was 4.58 per million per year (95% CI: 4.46–4.70). The incidence in females was lower than in males, with a rate ratio of 0.78 (95% CI: 0.74–0.83). For individuals 10–19 years old compared with those 1–9 years of age, IRR was 0.61 (95% CI: 0.57–0.64). NH White and NH Black cases had similar incidence rates. The IRRs for both Hispanic and NH API cases were significantly lower than NH White cases, at 0.87 (95% CI: 0.81–0.93) and 0.71 (95% CI: 0.62–0.82), respectively. By SES, the incidence was also higher for those who lived in counties in the top 25% compared with the middle 25–75% of counties.

**TABLE 1:** Incidence rate, incidence rate ratios, and average annual percent change of children and adolescents with rhabdomyosarcoma in the United States cancer statistics database, 2003–2017

Variable	No. (%)	IR	95% CI	IRR	95% CI	*p* value	AAPC	95% CI
Total	5656 (100)	4.58	4.46–4.70				0.3	−0.7–1.2
Sex								
Male	3236 (57)	5.12	4.95–5.30	Ref			0.2	−0.7–1.1
Female	2420 (43)	4.01	3.85–4.18	0.78	0.74–0.83	<0.0001	0.3	−1.3–2.0
Age at diagnosis (years)								
<1	323 (6)	5.39	4.82–6.02	0.94	0.84–1.05	0.30	0.9	−1.6–3.4
1–9	3123 (55)	5.74	5.54–5.94	Ref			0.3	−0.6–1.1
10–19	2210 (39)	3.48	3.34–3.63	0.61	0.57–0.64	<0.0001	0.2	−1.4–1.7
Race/Ethnicity								
NH White	3235 (57)	4.71	4.55–4.87	Ref			0.2	−0.8–1.2
NH Black	926 (16)	4.91	4.60–5.24	1.04	0.97–1.12	0.26	0.1	−2.0–2.2
NH API	215 (4)	3.36	2.93–3.84	0.71	0.62–0.82	<0.0001	2.7	0.0–5.5
NH AI/AN	53 (1)	4.22	3.16–5.52	0.90	0.67–1.18	0.47	—	—
NH All Other Races Combined	73 (1)	—	—	—	—	—	—	—
Hispanic	1153 (20)	4.10	3.86–4.34	0.87	0.81–0.93	<0.0001	−0.3	−2.0–1.4
Histology (ICD‐O‐3 code)								
Embryonal (ERMS)	2857 (51)	2.32	2.23–2.41	Ref			0.7	−0.3–1.7
Spindle	194 (3)	0.16	0.14–0.18	0.07	0.06–0.08	<0.0001	2.3	−2.3–7.0
Alveolar (ARMS)	1667 (29)	1.35	1.28–1.41	0.58	0.55–0.62	<0.0001	−0.9	−2.4–0.7
Others^a^	938 (17)	0.76	0.71–0.81	0.33	0.30–0.35	<0.0001	0.5	−1.5–2.6
Primary tumor site								
Favorable								
Orbit	375 (7)	0.31	0.28–0.34	Ref			0.6	−2.3–3.5
Genitourinary system	693 (12)	0.56	0.52–0.60	1.81	1.59–2.06	<0.0001	−1.2	−3.3–1.0
Biliary tract/liver	232 (4)	0.19	0.17–0.22	0.62	0.52–0.73	<0.0001	1.0	−2.6–4.7
Head/neck	1083 (19)	0.88	0.83–0.94	2.86	2.54–3.23	<0.0001	0.9	−1.2–3.1
Unfavorable								
Bladder/prostate	322 (6)	0.26	0.23–0.29	0.84	0.72–0.98	0.03	−2.2	−5.4–1.1
Para‐meningeal region	477 (8)	0.39	0.35–0.42	1.26	1.10–1.44	0.001	−2.9	−4.6–−1.2
Extremity	738 (13)	0.60	0.56–0.64	1.94	1.71–2.21	<0.0001	0.3	−1.8–2.4
Trunk/other	1715 (30)	1.39	1.32–1.45	4.50	4.02–5.05	<0.0001	1.7	0.6–2.8
Unknown	20 (<1)	0.02	0.01–0.03	0.05	0.03–0.08	<0.0001	—	—
Stage								
Localized	1923 (34)	1.56	1.49–1.63	Ref			1.3	0.1–2.4
Regional	1831 (32)	1.48	1.42–1.55	0.95	0.89–1.01	0.12	−0.9	−2.4–0.8
Distant	1632 (29)	1.32	1.25–1.38	0.84	0.79–0.90	<0.0001	0.6	−0.6–1.9
Unknown	270 (5)	0.22	0.19–0.25	0.14	0.12–0.16	<0.0001	−1.4	−6.3–3.7
US Census region								
Northeast	1007 (18)	4.90	4.60–5.21	Ref			0.6	−1.3–2.5
Midwest	1220 (22)	4.55	4.30–4.81	0.93	0.85–1.01	0.09	0.7	−0.9–2.4
South	2112 (37)	4.56	4.37–4.76	0.93	0.86–1.01	0.07	0	−0.9–1.0
West	1317 (23)	4.43	4.19–4.67	0.90	0.83–0.98	0.02	−0.1	−1.4–1.2
Socioeconomic status								
Top 25%	1597 (28)	4.82	4.58–5.06	Ref			0.5	−0.9–2.0
25% ‐ 75%	3243 (57)	4.49	4.34–4.65	0.93	0.88–0.99	0.02	0.2	−0.8–1.1
Bottom 25%	653 (12)	4.48	4.15–4.84	0.93	0.85–1.02	0.13	−0.1	−1.5–1.4
Unknown	163 (3)	—	—	—	—	—	—	—
Population density by county								
Metro (population)	4922 (87)	4.77	4.64–4.90				0.3	−0.7–1.3
>1,000,000	3244 (57)	4.89	4.72–5.06	Ref			0.5	−0.9–1.8
250,000‐1,000,000	1186 (21)	4.53	4.28–4.80	0.93	0.87–0.99	0.03	−0.2	−1.9–1.6
<250,000	492 (9)	4.61	4.21–5.04	0.94	0.86–1.04	0.24	0.6	−1.3–2.5
Non‐Metro	731 (13)	4.34	4.03–4.66	0.89	0.82–0.96	0.003	−0.1	−1.4–1.3

*Note:* Dash indicates unable to calculate due to sampling size/missing data. Abbreviations: AAPC, average annual percent change; AI/AN, American Indian/Alaska Native; API, Asian‐Pacific Islander; CI, confidence interval; ICD, International Classification of Diseases; IR, incidence rate; IRR, incidence rate ratio; NH, non‐Hispanic; SEER, Surveillance, Epidemiology, and End Results.

^a^ Other histologies include RMS NOS, mixed‐type RMS, and embryonal sarcoma. Variables with missing individual cases (n): Race/Ethnicity (1), Primary tumor site group (1), Population density (3).

ERMS was the most common histologic subtype (51%), followed by ARMS (29%). The most common primary tumor site was trunk/other (30%), followed by head/neck (19%), extremity (13%), and genitourinary system (12%). The SEER stage was relatively evenly distributed among all cases. The incidence of RMS overall did not change significantly during 2003–2017 (Table 1, AAPC 0.3, 95% CI: −0.7 to 1.2), nor were their significant changes when stratified by histology, with ERMS having an AAPC 0.7 (95% CI: −0.3 – 1.7) and ARMS having an AAPC ‐0.9 (95% CI: −2.4 to 0.7). Likewise, incidence did not change by sex, age, SES, population density, or geographic region. There was an increase for patients with localized disease (AAPC 1.3, 95% CI: 0.1–2.4), however, while other SEER stages showed stable incidence.

### 3.2 Survival

**FIGURE 1:**
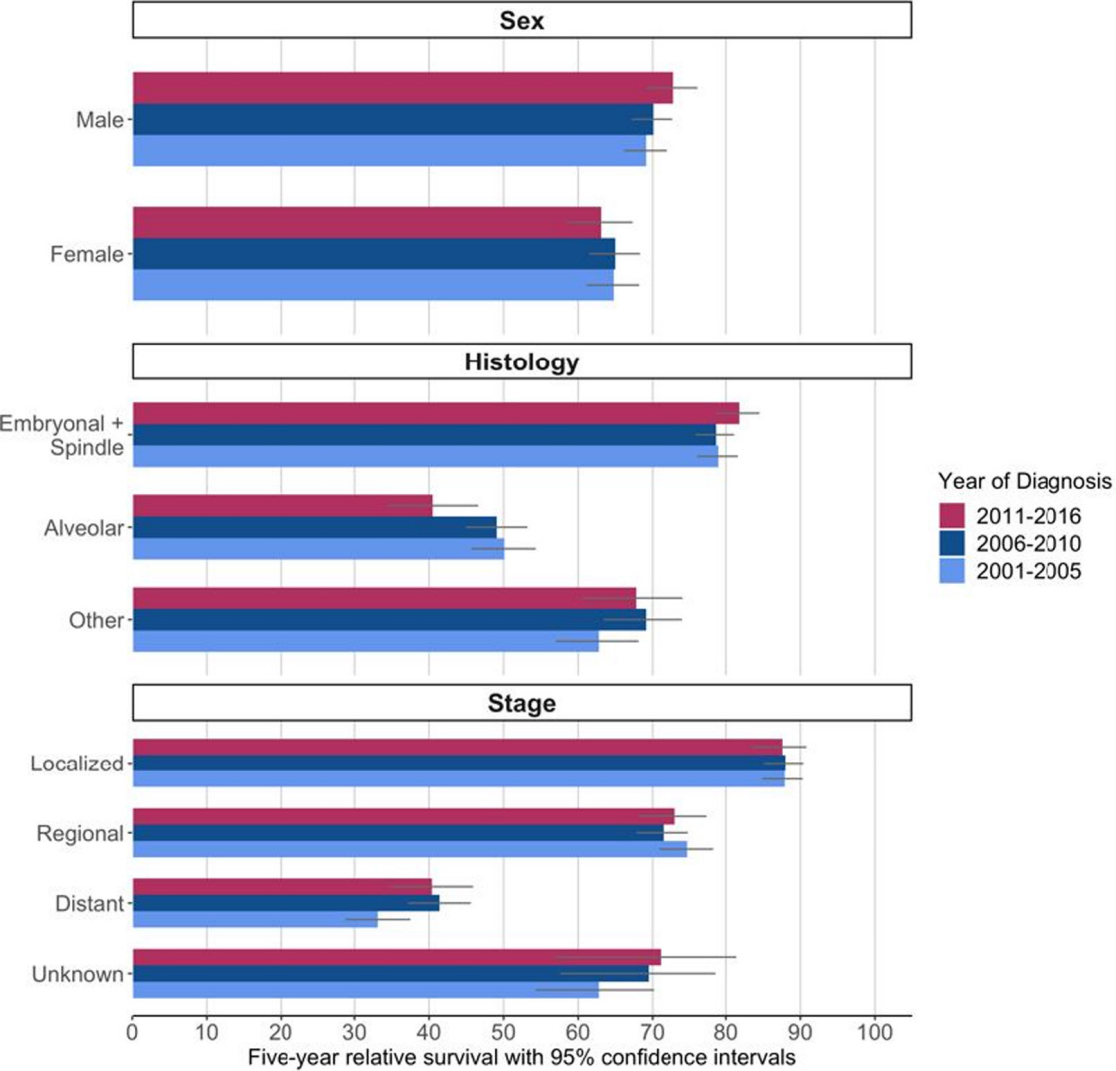
Relative survival for children and adolescents with rhabdomyosarcoma by year of diagnosis for sex, histology, and stage.

In the NPCR database, for the period 2001–2016, 5589 cases of RMS diagnosed in children and adolescents <20 years of age were identified. Overall, relative survival was 68.0% (95% CI: 66.6–69.3) (Table 2). RS in females (64.7%, 95% CI: 62.5–66.7%) was lower than in males (70.4%, 95% CI: 68.7–72.1%). Of the race/ethnicity groups with >200 patient cases, NH White cases showed the highest survival at 69.3% (95% CI: 67.6–71.0%), which was higher than NH Black cases at 64.4% (95% CI: 61.0–67.6%). Similarly, cases identified in counties with the highest SES (69.6%, 95% CI: 66.9–72.1%) had higher survival than those living in the lowest SES areas (65.5%, 95% CI: 61.6–69.1%), but confidence intervals overlapped. Relative survival was unchanged across time intervals when comparing 95% confidence intervals (Figure 1). Notably, there may be a widening trend over time in survival difference by sex, with the 2011–2016 period having the largest discrepancy (males [*n* = 1173]: 72.9%, 95% CI: 69.2–76.2%; females [*n* = 900]: 63.1%, 95% CI: 58.5–67.3%). No other evaluated variables suggest any definitive temporal changes in relative survival.

**TABLE 2:** Five‐year relative survival (RS) and univariate all‐cause survival hazard ratios (HR) for children and adolescents with rhabdomyosarcoma in the national program of cancer registries, 2001–2016

		Relative survival		All‐cause survival		
Variable	No. (%)	5‐year RS (%)	95% CI (%)	HR	95% CI	*p* value
Total	5589 (100)	68.0	66.6–69.3			
Sex						
Male	3228 (58)	70.4	68.7–72.1	Ref		
Female	2361 (42)	64.7	62.5–66.7	1.25	1.13–1.38	<0.0001
Age at diagnosis (years)						
<1	317 (6)	70.6	64.8–75.7	1.45	1.16–1.82	0.001
1–9	3094 (55)	77.2	75.5–78.7	Ref		
10–19	2178 (39)	54.4	52.1–56.6	2.30	2.07–2.55	<0.0001
Race/Ethnicity						
NH White	3269 (58)	69.3	67.6–71.0	Ref		
NH Black	941 (17)	64.4	61.0–67.6	1.20	1.05–1.36	0.01
NH API	196 (4)	64.8	56.7–71.8	—	—	—
NH AI/AN	53 (1)	69.9	53.3–81.5	—	—	—
Hispanic	1130 (20)	67.5	64.5–70.4	1.09	0.96–1.24	0.18
Histology (ICD‐O‐3 code)						
Embryonal (ERMS)	2802 (50)	79.2	77.5–80.7	Ref		
Spindle	197 (4)	85.4	79.0–90.0	0.66	0.44–0.99	0.04
Alveolar (ARMS)	1653 (30)	47.9	45.2–50.5	2.96	2.64–3.31	<0.0001
Others^a^	937 (17)	66.9	63.5–70.0	1.76	1.52–2.04	<0.0001
Primary tumor site						
Favorable						
Orbit	364 (7)	93.4	90.0–95.7	Ref		
Genitourinary system	711 (13)	87.4	84.5–89.8	2.07	1.28–3.34	0.003
Biliary tract/liver	230 (4)	82.8	76.8–87.4	3.05	1.77–5.23	<0.0001
Head/neck	1066 (19)	71.9	68.8–74.7	5.00	3.20–7.80	<0.0001
Unfavorable						
Bladder/prostate	326 (6)	74.4	68.9–79.1	4.44	2.74–7.20	<0.0001
Para‐meningeal region	493 (9)	62.8	58.1–67.1	6.93	4.40–10.90	<0.0001
Extremity	744 (13)	54.7	50.7–58.6	8.62	5.54–13.43	<0.0001
Trunk/other	1633 (29)	55.4	52.7–58.0	8.96	5.80–13.84	<0.0001
Unknown	22 (<1)	36.4	17.4–55.7	N/A	—	—
Stage						
Localized	1892 (34)	87.9	86.2–89.4	Ref		
Regional	1801 (32)	73.0	70.7–75.1	2.45	2.07–2.90	<0.0001
Distant	1575 (28)	38.5	35.9–41.1	7.55	6.46–8.82	<0.0001
Unknown	321 (6)	66.9	61.0–72.0	N/A	—	—
US Census region						
Northeast	988 (18)	68.6	65.4–71.6	Ref		
Midwest	1060 (19)	69.9	66.9–72.8	0.95	0.81–1.13	0.57
South	2183 (39)	67.6	65.4–69.7	1.03	0.90–1.19	0.66
West	1358 (24)	66.6	63.8–69.2	1.08	0.93–1.26	0.32
Socioeconomic status						
Top 25%	1480 (26)	69.6	66.9–72.1	Ref		
25%–75%	3283 (59)	67.6	65.8–69.3	1.12	0.99–1.26	0.07
Bottom 25%	716 (13)	65.5	61.6–69.1	1.17	0.99–1.38	0.07
Unknown	110 (2)	74.2	64.1–81.8	0.83	0.56–1.25	0.37
Population density by county						
Metro (population)	4884 (87)	67.9	66.4–69.3			
>1,000,000	3299 (59)	68.4	66.7–70.1	Ref		
250,000‐1,000,000	1119 (20)	66.9	63.8–69.8	1.08	0.95–1.22	0.26
<250,000	466 (8)	66.1	61.3–70.5	1.13	0.95–1.35	0.17
Non‐Metro	704 (13)	68.7	64.9–72.2	1.00	0.86–1.17	0.99
Year of diagnosis						
2001–2005	1712 (31)	67.4	65.1–69.5	Ref		
2006–2010	1804 (32)	67.9	65.7–70.0	0.96	0.86–1.08	0.53
2011–2016	2073 (37)	68.6	65.7–71.2	0.94	0.83–1.06	0.31

*Note:* Dash indicates unable to calculate due to sampling size/missing data. Abbreviations: AI/AN, American Indian/Alaska Native; API, Asian‐Pacific Islander; CI, confidence interval; HR, Cox hazard ratio; ICD, International Classification of Diseases; NH, non‐Hispanic; RS, relative survival; SEER, surveillance, epidemiology, and end results.

^a^ Other histologies include RMS NOS, mixed‐type RMS, and embryonal sarcoma. Patients with NH unknown race have been excluded from this analysis (*n* = 63). Variables with missing individual cases (n): population density (1).

**FIGURE 2:**
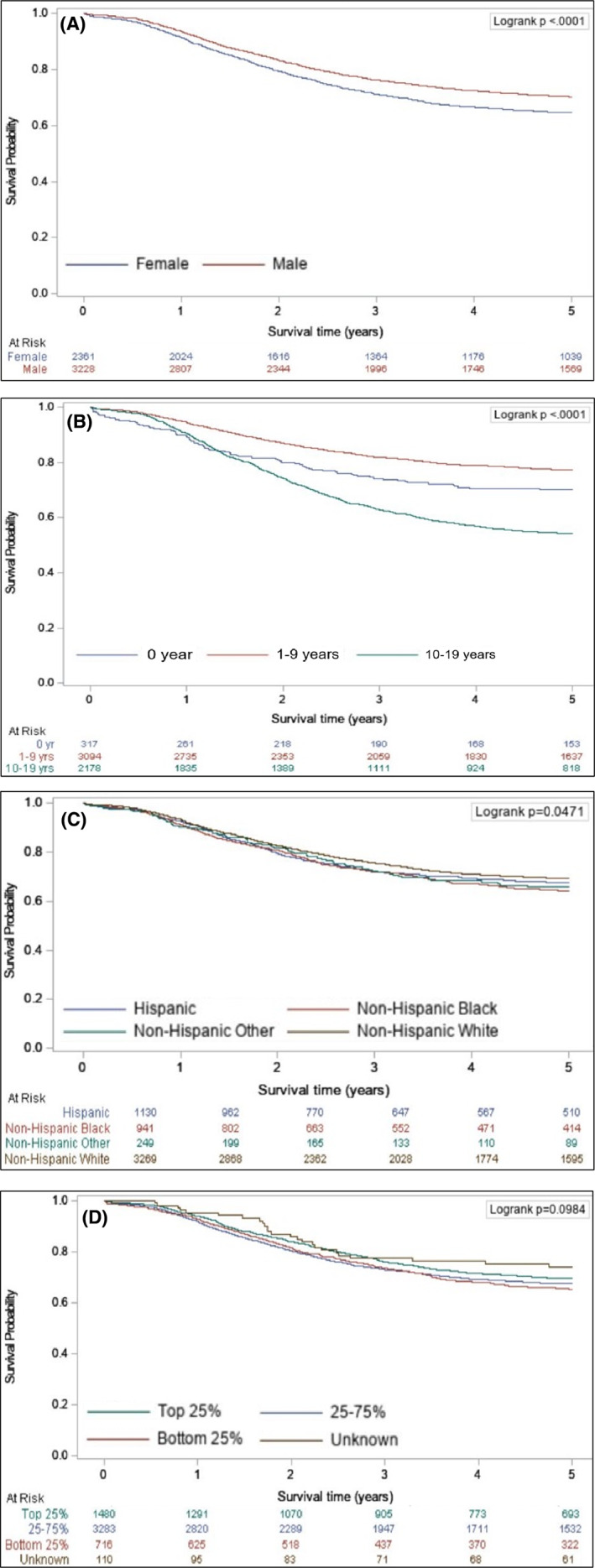
Kaplan–Meier survival estimation curve for children and adolescents with rhabdomyosarcoma by (A) sex, (B) age, (C) race/ethnicity, and (D) socioeconomic status.

Using Kaplan–Meier Survival curves, significant differences in survival were seen for sex (Figure 2A), age (Figure 2B), race/ethnicity (Figure 2C), anatomic site, histology, and stage (Figure S1). Cases with 1–9 years of age at diagnosis, orbital or genitourinary anatomic site, spindle cell or embryonal tumors, and localized disease all had the highest survival. Significant differences in survival curves were not seen for SES, year of diagnosis, population density by county, and US Census region.

**FIGURE 3:**
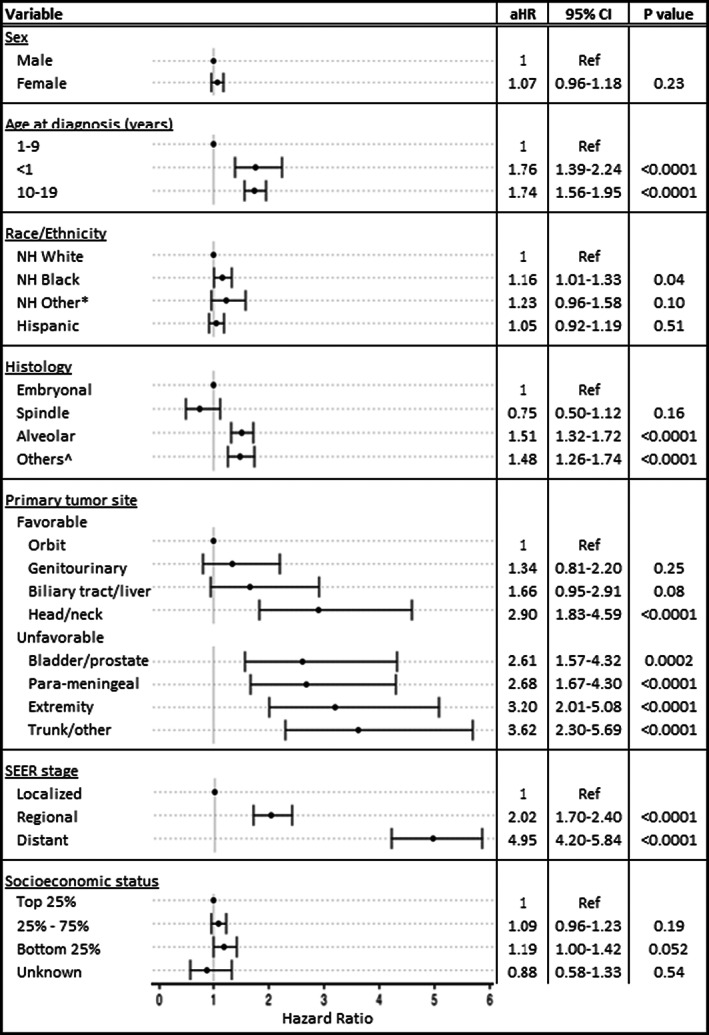
Multivariable Cox regression analysis with adjusted hazard ratios (aHR) for 5‐year all‐cause survival in children and adolescents with rhabdomyosarcoma in the National Program of Cancer Registries, 2001–2016. *Patients with NH Asian‐Pacific Islander and NH American Indian/Alaska Native race are combined into a single variable (NH Other). NH Unknown race has been excluded from this analysis (*n* = 63). ^Other histologies include RMS NOS, mixed‐type RMS, and embryonal sarcoma. aHR, adjusted hazard ratio; NH, non‐Hispanic; SEER, Surveillance, Epidemiology, and End Results.

In the multivariable analysis of 5‐year all‐cause survival, age <1 year or 10–19 years, ARMS histology, unfavorable anatomic site, and regional or distant stage were associated with poor survival (aHR >1.50, *p* < 0.001 for all) compared with the referent (Figure 3). NH Black cases had a 16% increased risk of death (95% CI: 1.01–1.33, *p* = 0.04) compared with NH White cases. Finally, cases identified in areas with relatively lower‐income levels had a 19% higher risk of death (95% CI: 1.00–1.42, *p* = 0.052) compared with those living in areas with higher‐income levels.

## 4 DISCUSSION

In one of the largest population‐based studies of pediatric RMS including over 5000 cases, we found stable incidence overall and by most demographic and clinical variables for the period 2003–2017. We also did not observe an improvement in 5‐year RS for the 2001–2016 study period, which is consistent with other epidemiological reports^7^, ^16^, ^20^ and COG clinical trials.^3^, ^15^, ^17^, ^18^, ^19^ While we confirmed the role of multiple previously identified prognostic factors – such as age, stage, histology, and tumor site^4^, ^7^ – on survival, we also observed a novel association for race.

In our study, the incidence of RMS overall has been stable over the past two decades, which contrasts with increasing trends found in several other extracranial solid tumors such as thyroid carcinomas and renal and hepatic tumors.^37^, ^38^ We observed stable incidence rates for individual histologic subtypes, contrasting with two previous reports with smaller sample sizes using SEER data which showed increasing incidence for ARMS tumors diagnosed before 2006.^16^, ^20^ Our disparate result is likely explained by shifts in diagnostic criteria for ARMS histology in the United States in the 1990s and early 2000s, followed by the widespread adoption of more precise and comprehensive testing using immunohistochemistry and fluorescent in situ hybridization (FISH), as recently described by Rudzinski et al.^29^

We observed significant differences in incidence by race/ethnicity and other sociodemographic factors. For example, we found the incidence of RMS was significantly lower in Hispanic and API cases compared with NH White cases, which is consistent with other studies.^14^, ^16^ Additionally, we observed a higher incidence among those living in higher SES areas. The few studies evaluating the role of SES on RMS have been equivocal, with both direct and inverse associations reported.^39^, ^40^ Ultimately, more targeted studies can help understand the different causal factors that may be driving these observed differences and potential strategies to reduce RMS incidence.

We observed a small but statistically significant difference in survival by race. Specifically, we found that NH Black cases with RMS had worse survival compared with NH White cases. Previous studies have produced inconsistent results in this area. Pui et al. conducted one of the earliest explorations of pediatric outcomes by race and found no differences in 5‐year survival in their regional cohort (patients diagnosed 1962–1992; RMS patients, *n* = 289).^10^ Using Intergroup Rhabdomyosarcoma Study Group data from multiple clinical trials, Baker et al. also found no racial differences in 5‐year failure‐free survival, despite Black patients often having higher‐risk disease features (1983–1997; *n* = 2350).^11^ Using different iterations of the SEER database, Ognjanovic et al. observed improved RS for Black patients (SEER‐9, 1996–2000; *n* = 166), whereas Johnson et al. reported worse outcomes by ethnicity, but not by race (SEER‐17, 1985–2005; *n* = 1228).^13^, ^16^ Importantly, none of these studies had available SES data to address confounding. More recently, Kehm et al. performed a mediation method analysis and reported an unadjusted mortality HR of 1.44 (95% CI: 1.10–1.88) for Black children and adolescents that did not hold after accounting for SES (SEER 18, 2000–2011; *n* = 1202).^41^ Given our study's sample size and multivariable regression that controlled for SES, we provide the strongest evidence to date of this racial disparity in RMS outcomes. Such race‐/ethnicity‐based survival differences have been reported for many pediatric cancers and are largely considered multifactorial, with Pui et al. demonstrating that unequal access to care may be one major driving element regardless of cancer type.^42^, ^43^, ^44^ Furthermore, enrollment in national cooperative trials, which is known to improve outcomes but also harbor racial disparities among children, warrants attention for potential differences.^45^, ^46^, ^47^ However, data for RMS specifically are lacking. The most recently completed COG study for RMS, ARST0531, enrolled 62 Black patients out of 448 total (13.8%), similar to the proportion of NH Black cases in this study (16% of RMS cases in the USCS database).^18^

Survival for the lowest SES group was lower than survival for the highest SES group, although the differences were not statistically significant. Lower survival has been associated with lower SES status in many cancers across all ages.^48^, ^49^, ^50^ This is the first large‐scale population‐based study to explore two family residential variables (geographic region and population density) for children and adolescents with RMS, and we found that these do not appear to have a significant impact on outcomes. However, an additional exploration into other aspects of social and economic advantage/disadvantages, such as health insurance status, access to care, and availability of community support systems, may be needed to identify and address disparities in survival.

Our study must be considered in light of certain limitations. As with any registry‐based study, some clinical and biological data were not available, there is no central pathology review, and patient vital status can be compromised by lack of active follow‐up or incomplete reporting. Most significantly, tumor fusion status and therapy information (e.g., chemotherapy agents, local control timing and methods, trial enrollment) are known to affect survival but were unavailable in the NPCR database. Furthermore, evolving diagnostic definitions and ICD terminology can introduce disease misclassification.^28^, ^32^, ^51^ For example, embryonal sarcoma comprised the vast majority of cases with the liver anatomic site but was kept in the analysis for consistency of comparison to previous population‐based studies. Notably, on post hoc analyses, we excluded such patients (*n* = 185) and found no change in our major finding of survival disparity by race on the univariate (HR 1.19, 95% CI: 1.04–1.36, *p* = 0.01) or multivariable (HR 1.15, 95% CI: 1.00–1.33, *p* = 0.044) models or the log‐rank test (*p* = 0.039). With conventional SEER staging, our study does not align with the combinatorial risk stratification system used by the COG.^52^, ^53^ Despite these limitations, the overall trends for survival in our population‐based data match well with the aforementioned results reported from COG clinical trials, suggesting a high concordance of findings despite the methodological study differences.

Strengths of this study include the broad coverage of the U.S. population afforded by the USCS and NPCR databases. To our knowledge, this is the largest and most representative exploration to date of incidence and outcomes for pediatric RMS in the U.S. The sample size provides the necessary power for detecting true differences between variables, which can aid in clarifying risk factors and trends that have inconsistent findings in previous analyses. Furthermore, the population‐based nature of the sample maximizes generalizability, since inclusion is not limited to those who are eligible for and/or have access to enrollment in a clinical trial with a formalized treatment protocol. Similar population‐based studies in Europe report the overall incidence and outcome data that generally match our results, though analyses into race/ethnicity differences in Europe are lacking.^24^, ^25^ Finally, with the addition of new variables such as geographic region and population density, our analysis offers a comprehensive investigation of potential factors related to incidence and survival for pediatric RMS.

Our study used updated national cancer registry data to assess incidence and outcomes over the past 20 years for the pediatric RMS population. We provide the largest, most comprehensive population‐based analysis to date for pediatric RMS. While overall incidence and survival rates were stable over the study period, potential disparities in outcome by race/ethnicity, SES, sex, and other variables merit attention and future research. With such a large and representative sample, our study results can assist clinicians and researchers in their decision‐making regarding risk stratification for individual patients, clinical trial design, and public health outreach initiatives.

## AUTHOR CONTRIBUTIONS

Matthew T. McEvoy: conceptualization, methodology, project administration, formal analysis, writing—original draft, writing—review and editing. David A. Siegel: conceptualization, data curation, methodology, project administration, formal analysis, writing—review and editing. Shifan Dai: conceptualization, data curation, methodology, project administration, formal analysis, writing—review and editing. M. Fatih Okcu: conceptualization, methodology, project administration, formal analysis, writing—review and editing. Mark Zobeck: formal analysis, writing—review and editing. Rajkumar Venkatramani: formal analysis, writing—review and editing. Philip J. Lupo: conceptualization, methodology, project administration, formal analysis, writing—original draft, writing—review and editing. All authors participated in the manuscript final approval and agree to be accountable for all aspects of the work.

## ETHICS APPROVAL

The current study is not considered human subjects research as only de‐identified registry data were used.

## DISCLAIMER

The findings and conclusions in this report are those of the authors and do not necessarily represent the official position of the Centers for Disease Control and Prevention.

## Supporting information


Figure S1

## Data Availability

The data that support the findings of this study are available on request by contacting uscsdata@cdc.gov. [26] The data are not publicly available due to privacy and legal restrictions.
