# Supplementary material for: Pediatric rhabdomyosarcoma incidence and survival in the United States: An assessment of 5656 cases, 2001–2017
Source: Cancer Med. 2022 Sep 7;12(3):3644–56. doi: 10.1002/cam4.5211 (PMC9939205; doi:10.1002/cam4.5211)

**Appendix**

**Supplementary Figure A. Kaplan-Meier Survival Estimation Curve for**

**Children and Adolescents with Rhabdomyosarcoma**


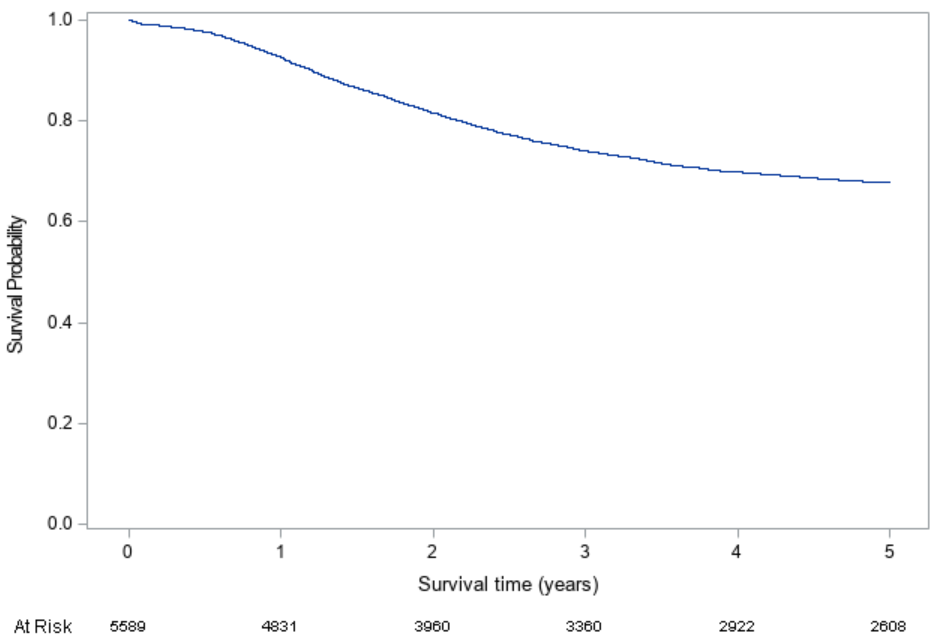


**Supplementary Figure B. Kaplan-Meier Survival Estimation Curve for**

**Children and Adolescents with Rhabdomyosarcoma by Primary Tumor Site**
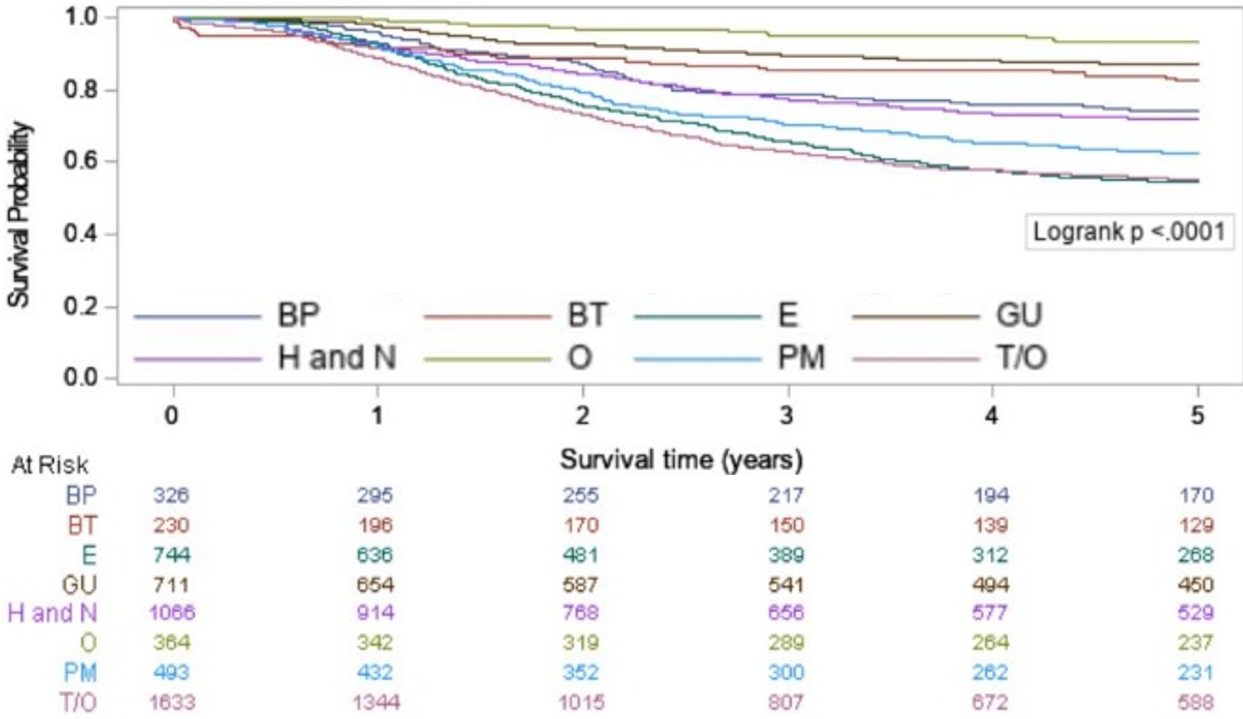


Abbreviations: BP, bladder/prostate; BT, biliary tract/liver; E, extremity; GU, genitourinary; H and N, head/neck;

O, orbit; PM, para-meningeal; T/O, trunk other.

**Supplementary Figure C. Kaplan-Meier Survival Estimation Curve for**

**Children and Adolescents with Rhabdomyosarcoma by Histology**
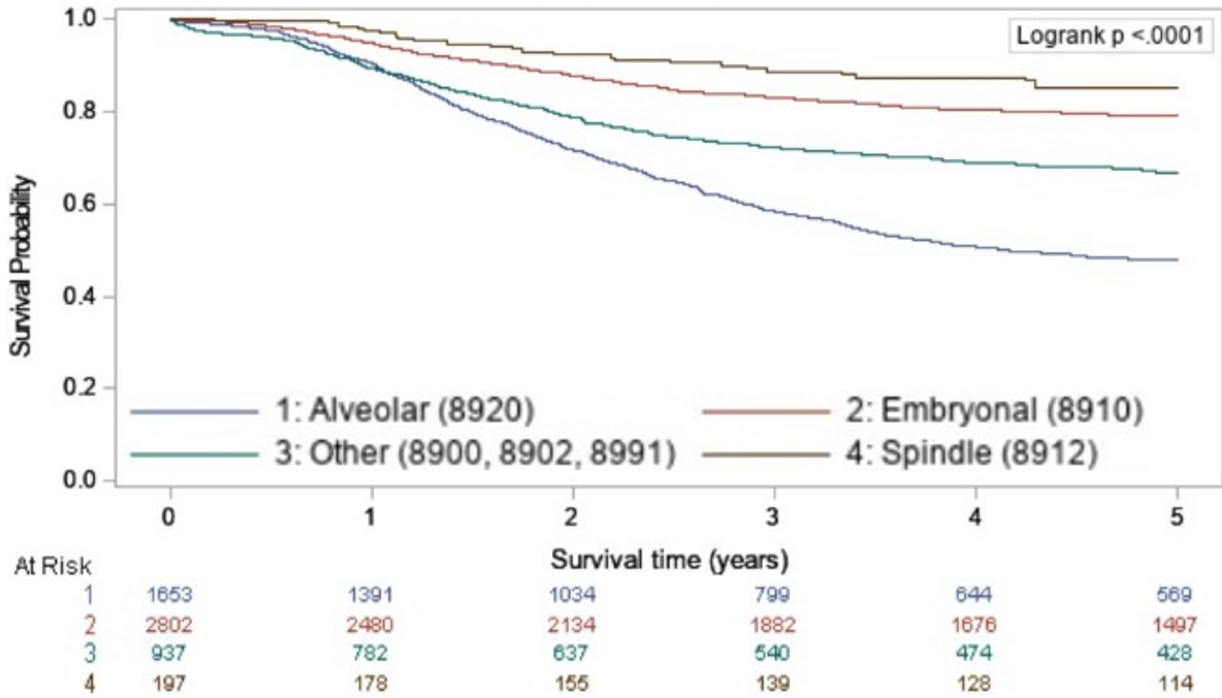


**Supplementary Figure D. Kaplan-Meier Survival Estimation Curve for**

**Children and Adolescents with Rhabdomyosarcoma by SEER Stage**
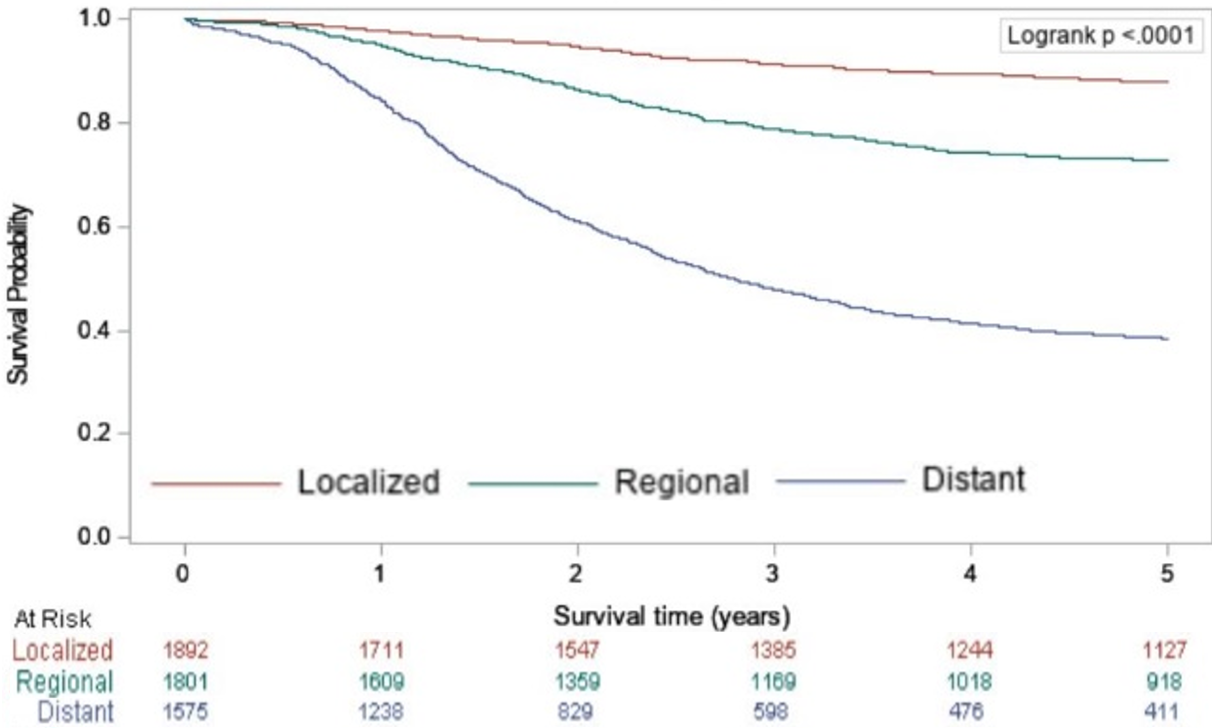


**Supplementary Figure E. Kaplan-Meier Survival Estimation Curve for**


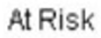
**Children and Adolescents with Rhabdomyosarcoma by Year of Diagnosis**
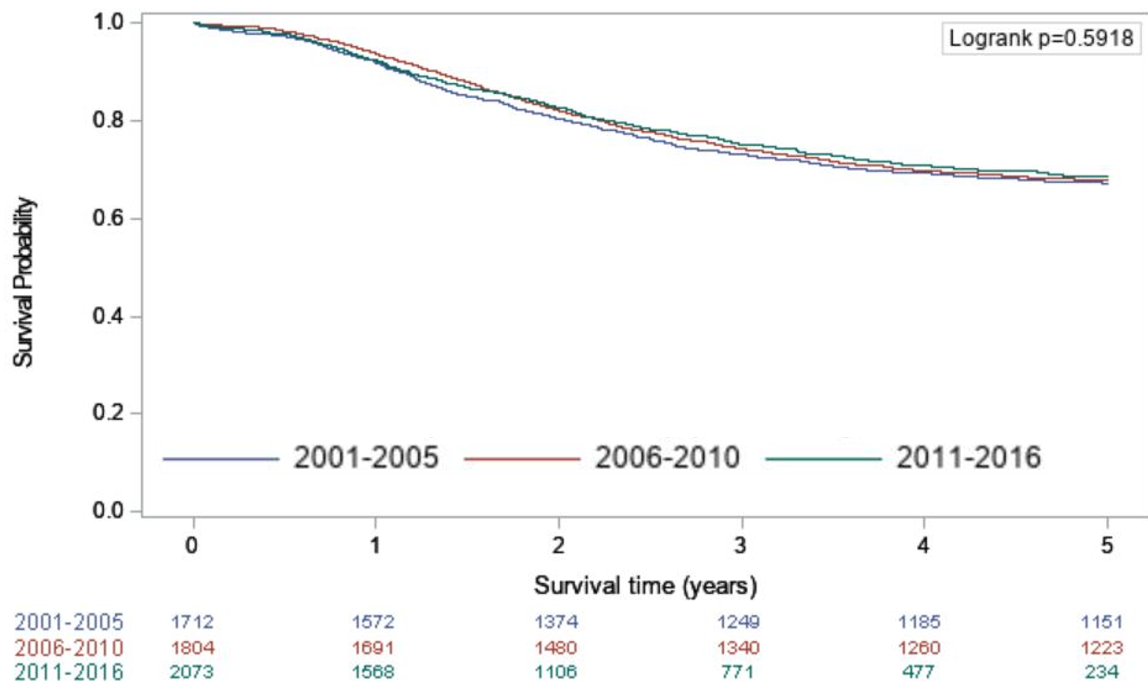


**Supplementary Figure F. Kaplan-Meier Survival Estimation Curve for**


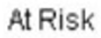
**Children and Adolescents with Rhabdomyosarcoma by Population Density by County**
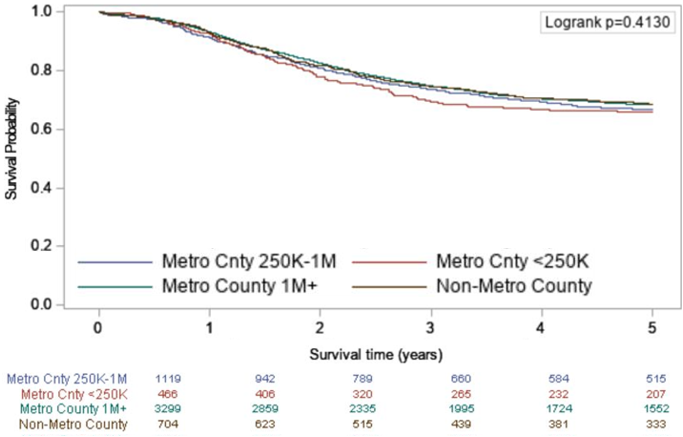


**Supplementary Figure G. Kaplan-Meier Survival Estimation Curve for**

**Children and Adolescents with Rhabdomyosarcoma by US Census Region**


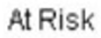

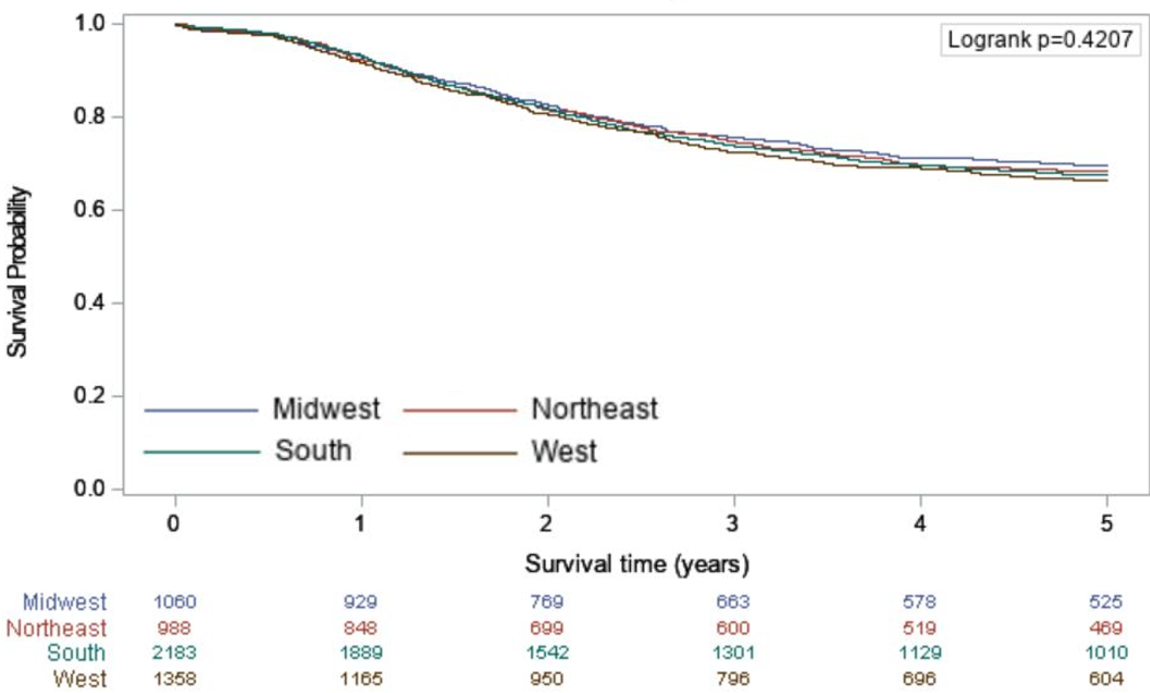

Supplement: Supplementary file 1 — Figure S1 [file CAM4-12-3644-s001.docx]
